# Stable fabrication of a large nanopore by controlled dielectric breakdown in a high-pH solution for the detection of various-sized molecules

**DOI:** 10.1038/s41598-019-49622-y

**Published:** 2019-09-11

**Authors:** Itaru Yanagi, Rena Akahori, Ken-ichi Takeda

**Affiliations:** Hitachi Ltd., Research & Development Group, Center for Technology Innovation - Healthcare, 1-280, Higashi-koigakubo, Kokubunji, Tokyo 185-8603 Japan

**Keywords:** Electronic properties and materials, Biosensors, Nanopores, Nanosensors

## Abstract

For nanopore sensing of various-sized molecules with high sensitivity, the size of the nanopore should be adjusted according to the size of each target molecule. For solid-state nanopores, a simple and inexpensive nanopore fabrication method utilizing dielectric breakdown of a membrane is widely used. This method is suitable for fabricating a small nanopore. However, it suffers two serious problems when attempting to fabricate a large nanopore: the generation of multiple nanopores and the non-opening failure of a nanopore. In this study, we found that nanopore fabrication by dielectric breakdown of a SiN membrane under high-pH conditions (pH ≥ 11.3) could overcome these two problems and enabled the formation of a single large nanopore up to 40 nm in diameter within one minute. Moreover, the ionic-current blockades derived from streptavidin-labelled and non-labelled DNA passing through the fabricated nanopore were clearly distinguished. The current blockades caused by streptavidin-labelled DNA could be identified even when its concentration is 1% of the total DNA.

## Introduction

Nanopores have been widely used in recent years as a highly sensitive microscope that can observe various biomolecules in an aqueous solution. The structural and electrical characteristics of the target molecules can be extracted by detecting the changes in ionic currents through the nanopore when the molecules pass through it. For instance, single-molecule DNA sequencing is currently possible^1–14^ by using a biological nanopore with a diameter of 2 nm or less. In addition, the detection of proteins, antigen-antibody complexes and probe-labelled DNA is possible^15–21^ by using a solid-state nanopore with a diameter of several to tens of nanometres. Moreover, the detection of various viruses is also possible^22–27^ by using a solid-state nanopore with a diameter of several tens to hundreds nanometers. To detect such varied molecules with high sensitivity, adjusting the size of the nanopore according to the size of each target molecule is important.

For solid-state nanopores, a nanopore with a diameter of a few to hundreds of nanometres can be fabricated by choosing an appropriate manufacturing process^28–38^. This capability is an advantage over biological nanopores whose diameters are typically limited to approximately 2 nm or less. A nanopore with a diameter of approximately 20 nm to several hundred nanometres can be fabricated by utilizing optical or electron-beam (EB) lithography followed by reactive-ion etching^29^. An even smaller nanopore down to 1 nm in diameter can be fabricated by drilling a membrane with a focused-electron or helium-ion beam^30–34^. In addition, dielectric breakdown of a membrane has been widely utilized as a method of nanopore fabrication in recent years^35–61^ because of its simplicity and inexpensiveness. With the application of a high constant voltage to a membrane and termination of the voltage when the current between the electrodes reaches a predetermined cut-off value, a nanopore can be created in a membrane. This method is called controlled breakdown (CBD)^35,36,39^, which also enables the fabrication of a small nanopore down to 1 nm in diameter.

CBD, however, suffers two problems for the fabrication of a single large nanopore with a diameter of larger than approximately 5 nm. One is the generation of multiple nanopores, which has been reported in several studies^40,41,43,44^. Our observed example of the generation of multiple nanopores in a 10-nm-thick SiN membrane is also shown in Supplementary Fig. 1. This behaviour is caused by the second and the subsequent dielectric breakdowns of the membrane occurring until the current reaches the cut-off current after the first dielectric breakdown (i.e., multiple nanopores generate while widening a nanopore generated at the first dielectric breakdown). When trying to fabricate a large nanopore, it is necessary to set a high cut-off current value. As a result, the time spent on the nanopore widening process increases, leading to a high risk of the generation of multiple nanopores.

This problem seems to be solved if the time of the nanopore widening process, compared to the time to the first dielectric breakdown, can be sufficiently shortened. In this context, a thicker membrane is expected to be suitable for the fabrication of a single large nanopore. A higher voltage is required to break down a thicker membrane. Consequently, a higher Joule energy is released after the first dielectric breakdown, which contributes to faster nanopore enlargement. However, the second problem appears here. As we reported previously^56^, when CBD was applied to a thick (20-nm-thick) SiN membrane, a nanopore could not be fabricated, and only a local conductive-film portion was created. With an increase in the cut-off current value, the area of the conductive-film portion expanded, and the membrane was eventually destroyed. Therefore, the fabrication of a single large nanopore by CBD has been very difficult.

Recently, it has been reported that the laser-assisted CBD can form a large nanopore with a diameter of 20–50 nm^57^. While this technique is revolutionary, it requires a laser optical system for nanopore fabrication. In this study, we report a method that enables the fabrication of a single large nanopore in a thick membrane by CBD without the need for optics. The application of CBD to a SiN membrane with a thickness of 20 nm or 14 nm in an aqueous solution with a pH higher than 11.3 enabled the fabrication of a nanopore instead of a conductive-film portion. The diameter of the fabricated nanopore could be roughly controlled within a range from 5 nm to 40 nm by changing the cut-off current value. In addition, the occurrences of streptavidin (SA)-labelled and non-labelled DNA passing through a nanopore were clearly discriminated by monitoring the difference in ionic-current blockades derived from those molecules. Even if the SA-labelled DNA was present in only 1% of the total DNA in an aqueous solution, its presence could be detected.

## Results

**Figure 1:**
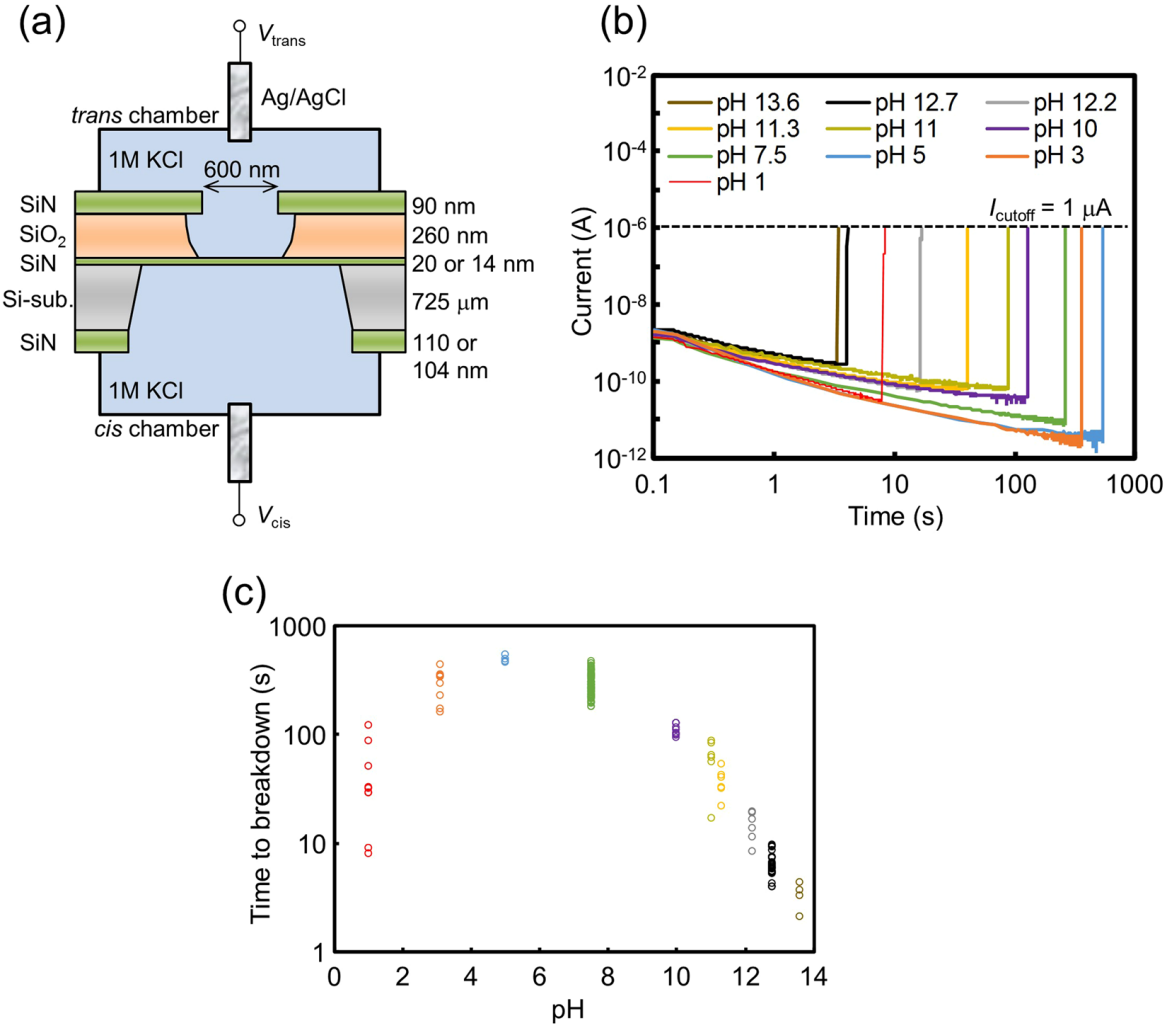
Dielectric breakdown of SiN membranes by CBD under various pH conditions. (**a**) Schematic illustration of the setup for dielectric breakdown experiments. (**b**) Current-time traces during CBD of 20-nm-thick SiN membranes under ten different pH conditions. *V*_cis_ and *V*_trans_ were set at 20 V and 0 V during CBD, respectively. (**c**) TBD of 20-nm-thick SiN membranes under the ten different pH conditions. *V*_cis_ and *V*_trans_ were set at 20 V and 0 V during CBD, respectively. The number of plots at each pH is at least four.

The schematic setup for the dielectric breakdown experiments is presented in Fig. 1(a). The area of the SiN membrane with a thickness of 20 nm or 14 nm was restricted within approximately a small square area of approximately 600 × 600 nm^2^ so that fabricated nanopores could be easily found. Two Ag/AgCl electrodes (*cis* and *trans* electrodes) were immersed in 1 M KCl aqueous solution at various pH values for applying voltages and measuring currents through the membrane. In this study, SiN membranes with thicknesses of 20 nm were mainly used for the dielectric breakdown experiments. Figure 1(b) presents examples of current-time traces when CBD was applied to 20-nm-thick SiN membranes under ten different pH conditions. The voltage applied was 20 V (*V*_trans_ = 0 V and *V*_cis_ = 20 V), and the cut-off current (*I*_cutoff_) was set at 1 μA. *I*_cutoff_ was set as a limit of the output current of the measuring instrument, and the voltage automatically dropped when the current between the electrodes reached *I*_cutoff_ ± 0.1%. The dielectric breakdown point was clearly confirmed in each time trace. Figure 1(c) presents the plots of the time to breakdown (TBD) under each pH condition. The TBD became shorter as the aqueous solution became more acidic or alkaline. In particular, faster breakdowns were prominently observed under alkaline conditions than under neutral or acidic conditions. Therefore, Si-N bond breakage in the membrane was thought to be strongly promoted by electrochemical reactions induced particularly by hydroxide ions. Notably, this trend is opposite to that reported by Kwok *et al*.^35^. According to their report, the TBD of SiN membranes at a pH of 2 was approximately 100 times shorter than that at a pH of 13.5. In addition, the longest TBD was observed at a pH of approximately 10 in their report, while it was observed at a pH of approximately 5 in our result. These differences are thought to be due to the difference in deposition conditions of the SiN membrane layers, which affects their basic material properties. Details of the formation process of our SiN membranes are described in the Methods section. Incidentally, the reason for shorter TBD under acidic condition is thought to be due to H^+^ or hole injection into the membrane which strongly promoted impact ionization-induced avalanche and Si-N bond breaking^35,39^.

**Figure 2:**
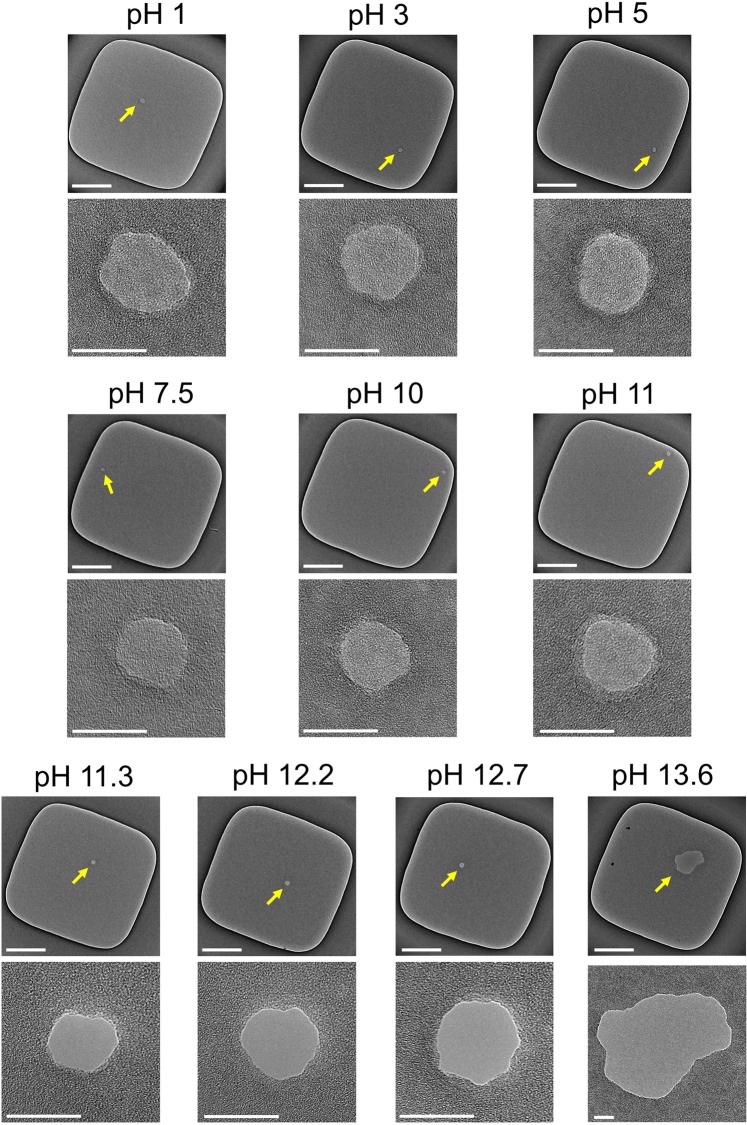
TEM images of SiN membranes after CBD under various pH conditions. An image of the entire membrane and a magnified view of the area around the created defect or nanopore are shown in each image set. Defective portions or nanopores are indicated by yellow arrows. The thickness of the SiN membranes was 20 nm, and *V*_cis_ and *V*_trans_ were set at 20 V and 0 V during CBD, respectively. *I*_cutoff_ was set at 1 μA. Scale bars for the images of the entire membranes are 200 nm, and those for the magnified views are 20 nm.

Figure 2 presents transmission electron microscopy (TEM) images of ten different 20-nm-thick SiN membranes after CBD under ten different pH conditions. During CBD, the applied voltage and *I*_cutoff_ were set at 20 V (*V*_trans_ = 0 V and *V*_cis_ = 20 V) and 1 μA, respectively. An image of the entire membrane and a magnified view of the area around the created defect are shown in each image set. From the entire images of the membranes, the number of created defects (indicated by yellow arrows) was confirmed to be one per membrane. The magnified views of the defects confirmed that there were two kinds of cases: instead of a nanopore, a local conductive-film portion was created (i.e., amorphous material was confirmed in the defect area), or a nanopore was created (i.e., no amorphous material was confirmed in the defect area). The conductive-film portion is thought to be a low-density film that allows ion conduction. We previously reported that only a conductive-film portion, not a nanopore, was created when CBD was applied to a 20-nm-thick SiN membrane at a pH of 7.5^56^. In the present experiment, the same result was obtained even when CBD was performed in a pH range from 1 to 11. On the other hand, interestingly, a nanopore was created when CBD was performed in the solution with a pH higher than 11.3. In addition, the size of the nanopore created increased as the pH of the solution increased. We subjected at least three membranes to CBD at each pH and confirmed the same trend described above. This result suggests that the membrane could be etched by hydroxide ions in the process of dielectric breakdown. Generally, it is known that SiN is hardly etched and Si is easily etched in an alkaline aqueous solution^62,63^. Therefore, the membrane was assumed to be etched by the following two steps: (i) Si-N bonds were destabilized by a high electric field during CBD, and the number of Si dangling bonds and Si-Si bonds increased. (ii) Si was etched by the following chemical reaction^62,63^:

1 $${\rm{Si}}+4{{\rm{OH}}}^{-}\to {\rm{Si}}{({\rm{OH}})}_{4}+4{{\rm{e}}}^{-},$$

2 $$2{{\rm{H}}}_{2}{\rm{O}}+{{\rm{2e}}}^{-}\to 2{\rm{OH}}+{{\rm{H}}}_{2}{\rm{.}}$$

**Figure 3:**
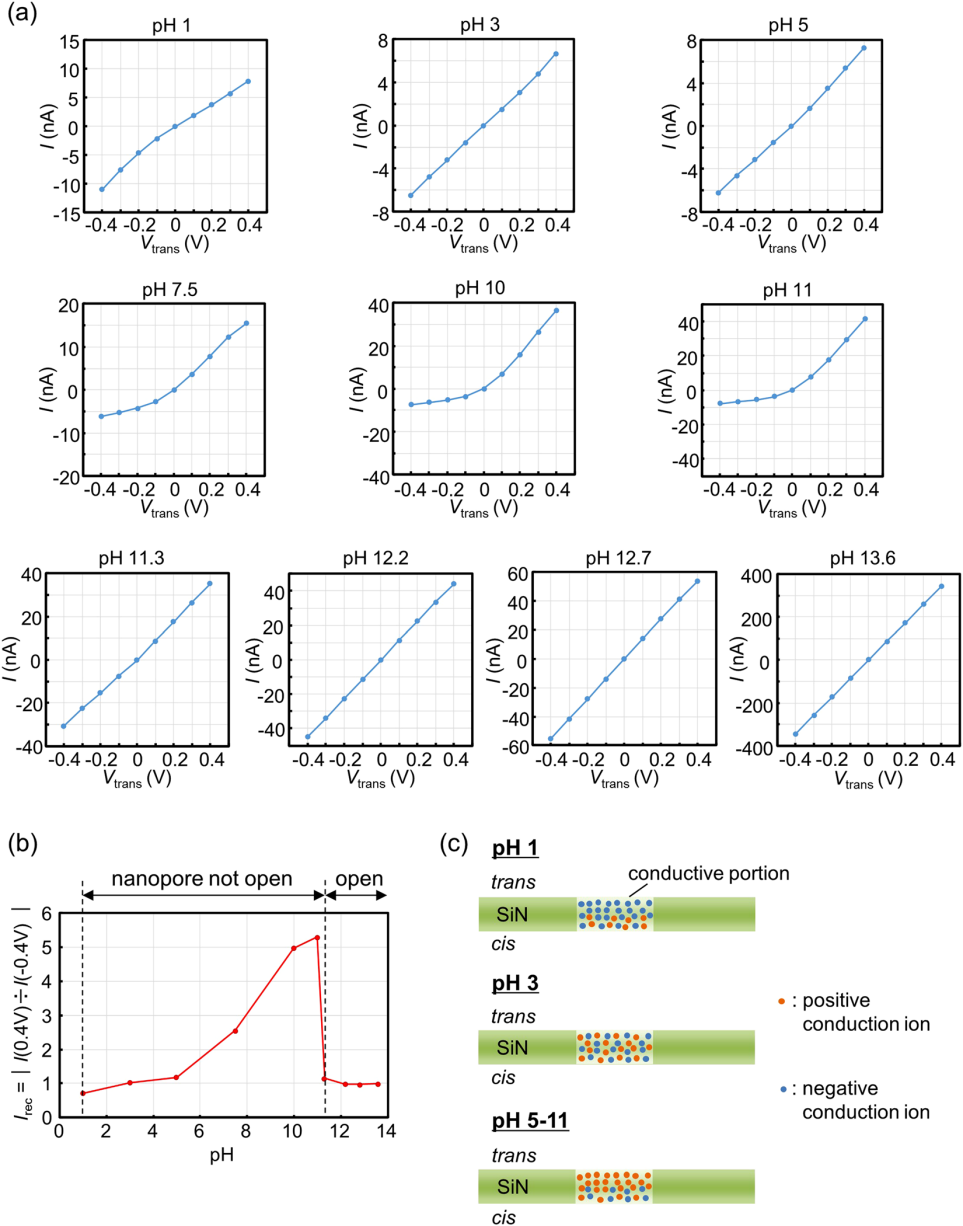
*I*-*V* characteristics of membranes after CBD under various pH conditions. (**a**) *I*-*V* characteristics of 20-nm-thick SiN membranes after CBD under ten different pH conditions. *V*_cis_ was set at 0 V during the measurements of the *I-V* curves. During CBD, *V*_cis_ and *V*_trans_ were set at 20 V and 0 V, respectively, and *I*_cutoff_ was set at 1 μA. (**b**) Dependence of *I*_rec_ on pH. (**c**) Schematic illustration of conduction-ion distribution in a conductive-film portion at each pH without voltage biases.

Figure 3(a) presents the corresponding *I*-*V* characteristics for each membrane after CBD. Figure 3(b) presents the pH dependence of *I*_rec_ = |*I*(0.4 V)/*I*(−0.4 V)|, which represents the degree and direction of each *I*-*V* curve’s rectification. In the case of pH ≥ 11.3 where a nanopore was created, each *I*-*V* curve showed almost ohmic characteristics, and *I*_rec_ was approximately 1. On the other hand, in the case of pH 1 to 11 where a nanopore was not created, the shape of the *I*-*V* curve and *I*_rec_ varied in accordance with the pH value. A convex upward *I*-*V* curve (i.e., *I*_rec_ < 1) at pH 1, an almost ohmic *I*-*V* curve (i.e., *I*_rec = _1) at pH 3, and convex downward *I*-*V* curves (i.e., *I*_rec_ > 1) at pH 5 to 11 were confirmed. *I*_rec_ monotonically increased with an increase in pH.

This result provided us the distribution of conduction carriers (i.e., conduction ions) in the conductive-film portion at each pH without voltage biases. Generally, a rectification of an *I*-*V* curve appears when there is an asymmetric carrier distribution in the direction of current flow. Semiconductor PN junction devices are the most well-known examples. Recently, it has been pointed out that such a rectification also occurs in a current through a conical nanopore^64,65^ or nanofluidic channel^66,67^ due to the asymmetric carrier distribution. Figure 3(c) presents the assumed conduction-ion distributions in the conductive-film portions. The density of negative conduction ions was thought to be high on the *trans* side of the conductive-film portion at pH 1 because *I*_rec_ < 1 and *V*_trans_ < *V*_cis_ was a forward-biased state. In the case of pH 3, *I*_rec_ was approximately 1, which suggested that there was almost no asymmetry in the distribution of conduction ions. On the other hand, the density of positive conduction ions was thought to be high on the *trans* side of the conductive-film portion at pH 5 to 11 because *I*_rec_ > 1 and *V*_trans_ < *V*_cis_ was a reverse-biased state. These positively and negatively biased carrier distributions were thought to be induced because the conductive-film portion itself was negatively or positively charged depending on the pH value; that is, the isoelectric point of the conductive-film portion was assumed to be approximately 3, and the portion was positively or negatively charged when the pH of the solution was smaller or larger than pH 3, respectively. Regarding the asymmetry of the conduction-ion distributions, the direction of the applied voltage during CBD determined which side (i.e., the *cis* or *trans* side) of the conductive-film portion the conduction ions tended to localize in. Accordingly, the directions of the rectifications reversed when CBD was performed with *V*_trans_ = 20 V and *V*_cis_ = 0 V (see Supplementary Fig. 2).

**Figure 4:**
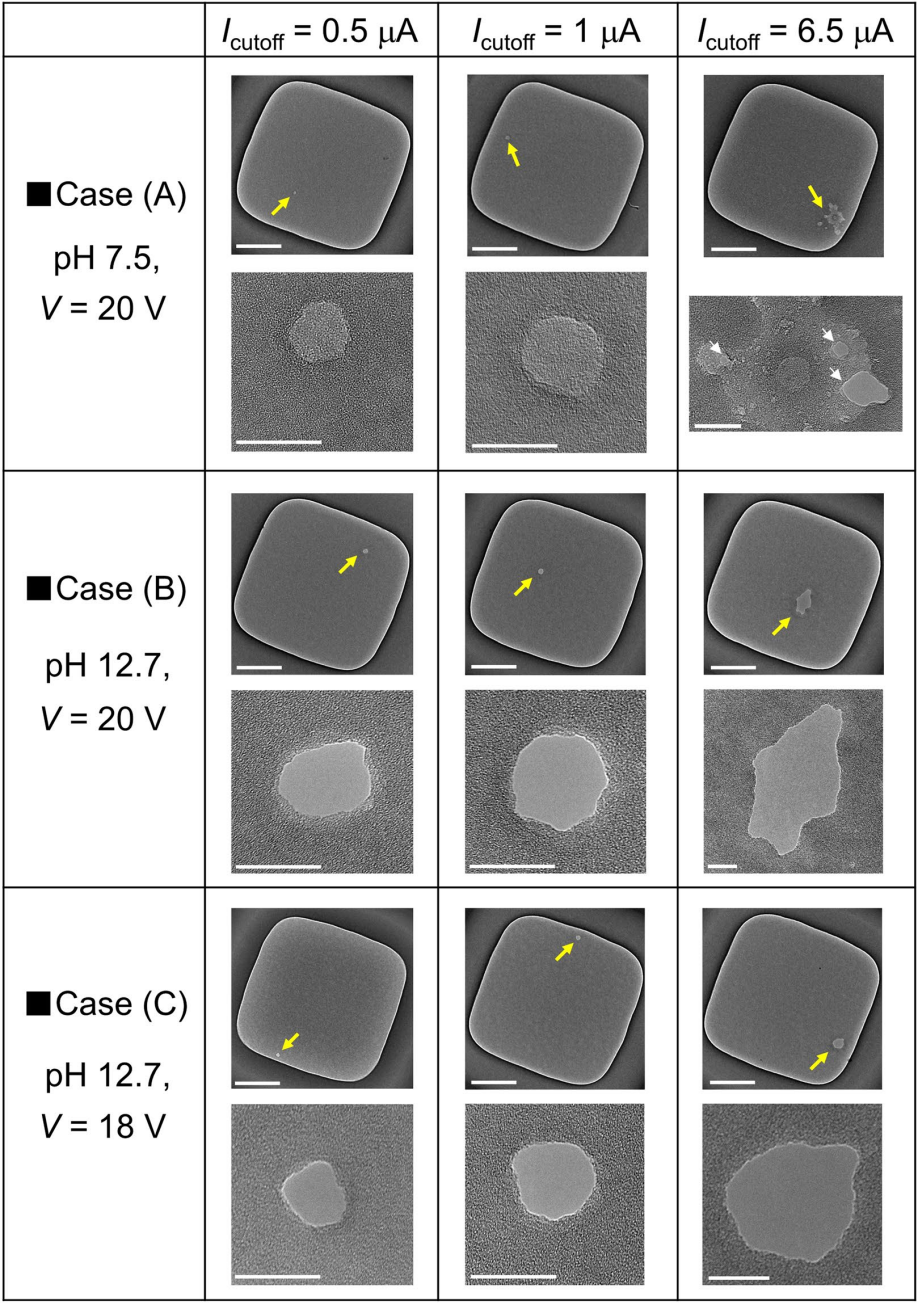
TEM images of SiN membranes after CBD under several pH, voltage, and *I*_cutoff_ conditions. An image of the entire membrane and a magnified view of the area around the created defect or nanopore are shown in each image set. Defective portions or nanopores are indicated by yellow arrows. The thickness of the SiN membranes was 20 nm. During CBD, *V*_cis_ was set at 20 V or 18 V, and *V*_trans_ was set at 0 V. Scale bars for the images of the entire membranes are 200 nm, and those for the magnified views are 20 nm.

Figure 4 presents TEM images of the nine different 20-nm-thick SiN membranes after CBD under several pH, voltage, and *I*_cutoff_ conditions. In case (A), where the pH was 7.5 and *V* = 20 V, the conductive-film portion became larger and multiple nanopores were generated as *I*_cutoff_ increased, which was reported in our previous study^56^. In case (B), where the pH was 12.7 and *V* = 20 V, a single nanopore was fabricated in each membrane regardless of the value of *I*_cutoff_. In addition, the size of the nanopore became larger or smaller with an increase or decrease in *I*_cutoff_, respectively. The nanopore size can also be controlled by changing the voltage. When the applied voltage was set at 18 V (i.e., in case (C)), the sizes of the created nanopores were comparatively smaller than those in case (B). The *I-V* characteristics for each membrane after CBD are presented in Supplementary Fig. 3, in which the behaviour of the curves was the same as the result in Fig. 3; that is, ohmic and rectified *I-V* curves were obtained for the nanopores and conductive-film portions, respectively.

**Figure 5:**
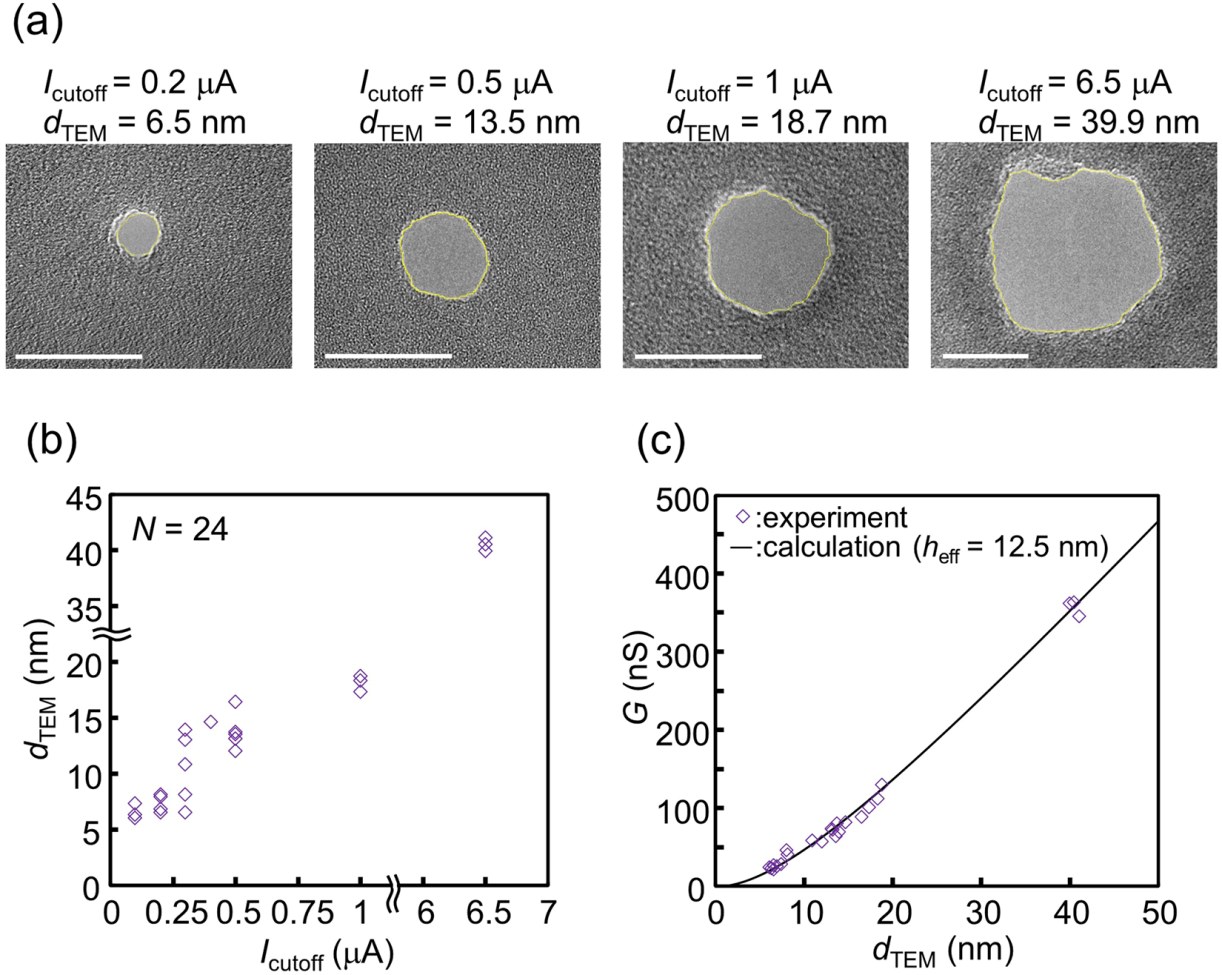
Dependence of the size of the fabricated nanopores on *I*_cutoff_. Nanopores were fabricated in 20-nm-thick SiN membranes by CBD at a pH of 12.7 with various *I*_cutoff_ values. *V*_cis_ and *V*_trans_ were set at 18 V and 0 V during CBD, respectively. (**a**) TEM images of the fabricated nanopores. Scale bars are 20 nm. The areas of the nanopores are surrounded by yellow lines. (**b**) Dependence of the equivalent diameter (*d*_TEM_) of the fabricated nanopores on *I*_cutoff_. (**c**) Relationship between conductance (*G*) of ionic current through the fabricated nanopores and *d*_TEM_. The solid line represents theoretically calculated values.

The dependence of the size of the created nanopore on *I*_cutoff_ was investigated in detail (Fig. 5). The nanopores were fabricated in 20-nm-thick SiN membranes by CBD at 18 V and pH 12.7. Each nanopore size was expressed by the equivalent diameter (*d*_TEM_), which was defined as:

3 $${d}_{{\rm{TEM}}}=2{(\frac{{S}_{{\rm{TEM}}}}{\pi })}^{1/2},$$

where *S*_TEM_ is the area of the nanopore surrounded by the yellow line in each TEM image, as shown in Fig. 5(a). The area was measured using image processing software (ImageJ). Figure 5(b) presents the relationship between *d*_TEM_ and *I*_cutoff_ (*N* = 24). Nanopores with a *d*_TEM_ of approximately 5 to 40 nm could be fabricated by changing the value of *I*_cutoff_ from 0.1 to 6.5 μA. Note that the number of nanopores observed was always one per membrane. Figure 5(c) presents the relationship between the conductance (*G*) of the ionic current through the nanopore and *d*_TEM_. The solid line represents the theoretically calculated relation assuming a cylindrical nanopore, which is obtained as follows^68–70^:

4 $$G=\sigma {(\frac{4{h}_{{\rm{eff}}}}{\pi {d}^{2}}+\frac{1}{d})}^{-1},$$

where σ = 0.123 S/cm is the measured conductance of a KCl aqueous solution with a pH of 12.7 at 24 °C, *d* is the diameter of the nanopore, and *h*_eff_ is the height of the nanopore (often called the effective thickness of the nanopore). The plotted measurements could be well fitted with the theoretically calculated line by setting *h*_eff_ at 12.5 nm. According to many previous reports^37,56,68–71^, *h*_eff_ is smaller than the actual membrane thickness. The result in this study is also consistent with this trend. The times required to form the nanopores by CBD were less than 1 min regardless of the size of the nanopore (see Supplementary Fig. 4(a)).

**Figure 6:**
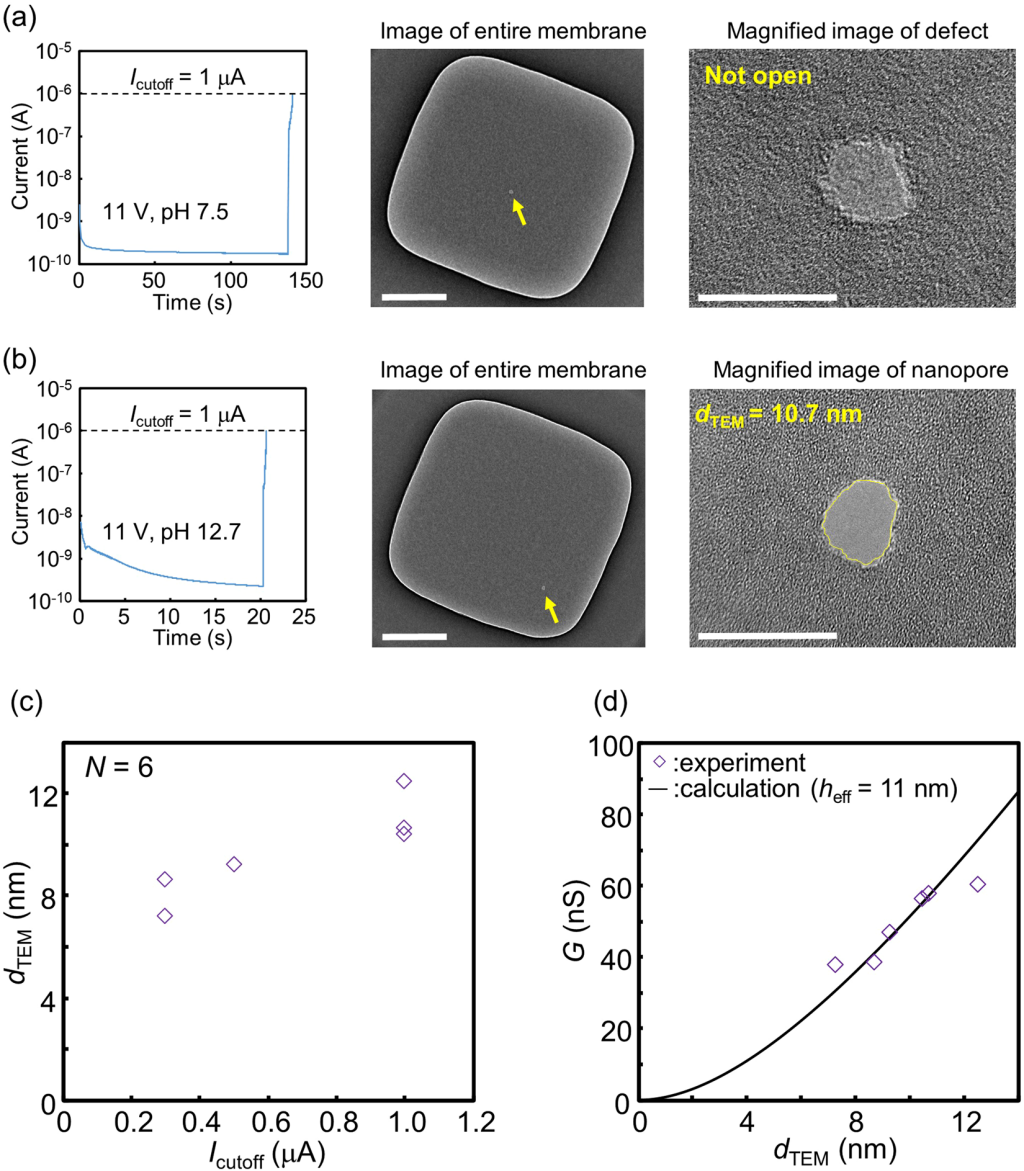
Nanopore fabrication in a 14-nm-thick SiN membrane by CBD under high-pH conditions. Current-time traces during CBD of SiN membranes, images of entire membranes after CBD and magnified views of created defects or nanopores when CBD was performed at (**a**) pH 7.5 and (**b**) pH 12.7. *V*_cis_ and *V*_trans_ were set at 11 V and 0 V, respectively, during CBD. *I*_cutoff_ was set at 1 μA. (**c**) Dependence of the equivalent diameter (*d*_TEM_) of the fabricated nanopores on *I*_cutoff_. (**d**) Relationship between conductance (*G*) of ionic current through the fabricated nanopores and *d*_TEM_. The solid line represents theoretically calculated values.

CBD under high-pH conditions was also effective for nanopore fabrication in 14-nm-thick SiN membranes (Fig. 6). The current-time traces during CBD and images of the membrane after CBD for each case at pH 7.5 and 12.7 are presented in Fig. 6(a,b). Similar to the case of the 20-nm-thick SiN membranes, CBD in a 14-nm-thick SiN membrane provided a conductive-thin portion instead of a nanopore at pH 7.5, whereas a nanopore was created in the case of pH 12.7. Figure 6(c) presents the dependence of *d*_TEM_ on *I*_cutoff_ (*N* = 6). All the nanopores were fabricated by CBD at an applied voltage of 11 V and pH of 12.7, and the *d*_TEM_ became larger with an increase in *I*_cutoff_. Figure 6(d) presents the relationship between *G* and *d*_TEM_. The effective thickness (*h*_eff_) was estimated to be approximately 11 nm by fitting the plotted measurements with Eq. (4). The times required to form the nanopores were also less than 1 min (see Supplementary Fig. 4(b)).

**Figure 7:**
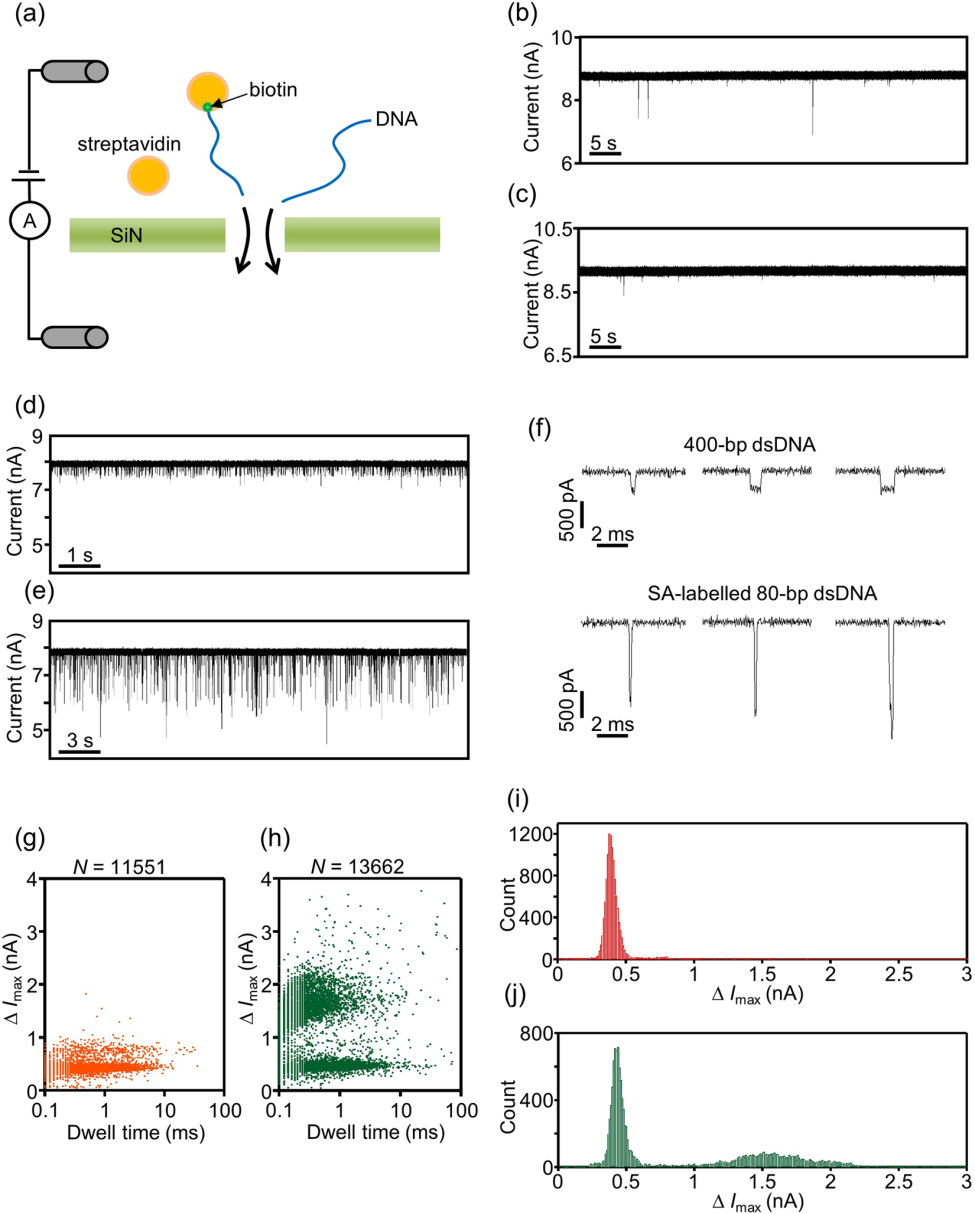
Detection of the passage of SA-labelled and non-labelled dsDNA through nanopores. (**a**) Schematic illustration of the detection of SA-labelled and non-labelled dsDNA passing through a nanopore. The voltage applied was 0.2 V (*V*_trans_ = 0.2 V and *V*_cis_ = 0 V). (**b**,**c**) Time traces of ionic current through nanopores fabricated in (**b**) 20-nm-thick and (**c**) 14-nm-thick SiN membranes. The solution in the *cis* chamber was 1 M KCl with 100 nM SA. The estimated diameters of the nanopores were (**b**) 10.4 nm and (**c**) 10.2 nm. (**d,e**) Time traces of ionic current through a nanopore fabricated in a 20-nm-thick SiN membrane. The solution in the *cis* chamber was (**d**) 1 M KCl with 15 nM 400-bp dsDNA. Then, the solution was incompletely displaced by (**e**) 1 M KCl with 15 nM SA-labelled 80-bp dsDNA. The estimated diameter of the nanopore was 9.8 nm. (**f**) Magnified views of typical ionic-current blockades derived from 400-bp dsDNA and SA-labelled 80-bp dsDNA passing through the nanopore. (**g,h**) Scatter plots of Δ*I*_max_ and dwell time for translocation events of (**g**) 400-bp dsDNA and (**h**) SA-labelled 80-bp dsDNA and 400-bp dsDNA. (**i,j**) Histograms of Δ*I*_max_ for translocation events of (**g**) 400-bp dsDNA and (**h**) SA-labelled 80-bp dsDNA and 400-bp dsDNA.

The nanopores fabricated in this study can accommodate large molecules such as labelled DNA. Therefore, we evaluated the sensing performance of the nanopores by examining whether the occurrences of streptavidin (SA)-labelled and non-labelled double-stranded DNA (dsDNA) passing through a nanopore could be discriminated on the basis of the difference in ionic-current blockades (Fig. 7(a)). After CBD of the membrane at a pH of 12.7, the solution in the *cis* and *trans* chamber was fully displaced by 1 M KCl aqueous solution at a pH of 8.7. Samples for the measurements were included in the *cis* chamber, and their passage was detected at an applied voltage of 0.2 V. The diameters of the fabricated nanopores were estimated by Eq. (4) with measured open-pore conductances. Biotin-streptavidin binding was utilized for preparing SA-labelled dsDNA (see the Methods section for details). The results of electrophoresis before and after labelling dsDNA with SA are presented in Supplementary Fig. 5. Biotin-modified dsDNA and SA were mixed at a ratio of 3:20 in the binding reaction. In the electropherograms of SA-labelled dsDNA, bands derived from remaining non-labelled dsDNA were also slightly observed. Consequently, when SA-labelled dsDNA was used for nanopore measurements, the sample also contained some non-labelled biotin-modified dsDNA. In this paper, we defined the description “x nM SA-labelled dsDNA” as the total molar concentration of SA-labelled and non-labelled biotin-modified dsDNA for the sake of simplicity.

To identify the current-blockade signals derived from SA-labelled dsDNA, free unbound SA should not pass through the nanopore. Therefore, we first examined whether free SA can pass through the nanopore. Figure 7(b,c) present respectively the time traces of ionic currents through the nanopores fabricated in the 20-nm- and 14-nm-thick SiN membranes when the aqueous solution in the *cis* chamber contained 100 nM SA. Almost no ionic-current blockades derived from SA translocation through the nanopores were confirmed (approximately less than 5 events/min). In the following experiments, the concentration of free SA in aqueous solution did not exceed 100 nM. Figure 7(d) presents the ionic current through the nanopore fabricated in the 20-nm-thick SiN membrane when the aqueous solution in the *cis* chamber contained 15 nM 400-bp dsDNA. Numerous ionic-current blockades caused by dsDNA translocations through the nanopore were observed. After the measurement, the solution in the *cis* chamber was partly displaced by the solution of 1 M KCl with 15 nM SA-labelled 80-bp dsDNA so that SA-labelled and non-labelled dsDNA could be mixed in the *cis* chamber. Figure 7(e) presents the ionic current through the nanopore after SA-labelled and non-labelled DNA were mixed in the *cis* chamber. Larger current blockades than those derived from dsDNA were frequently observed, which indicated the passage of SA-labelled dsDNA through the nanopore.

Magnified views of the typical current blockades when SA-labelled and non-labelled dsDNA passed through the nanopore are presented in Fig. 7(f). The dwell times of the current blockades derived from SA-labelled 80-bp dsDNA were typically shorter than those derived from 400-bp dsDNA, reflecting the difference in the dsDNA lengths. The scatter plots of the dwell time and Δ*I*_max_ and the corresponding histograms of Δ*I*_max_ before and after the mixture of SA-labelled and non-labelled dsDNA are presented in Fig. 7(g–j). Δ*I*_max_ was determined as the maximum current-blockade value observed in each current-blockade event. The figures confirmed that the occurrences of SA-labelled and non-labelled dsDNA passing through the nanopore could be clearly distinguished based on the value of Δ*I*_max_.

**Figure 8:**
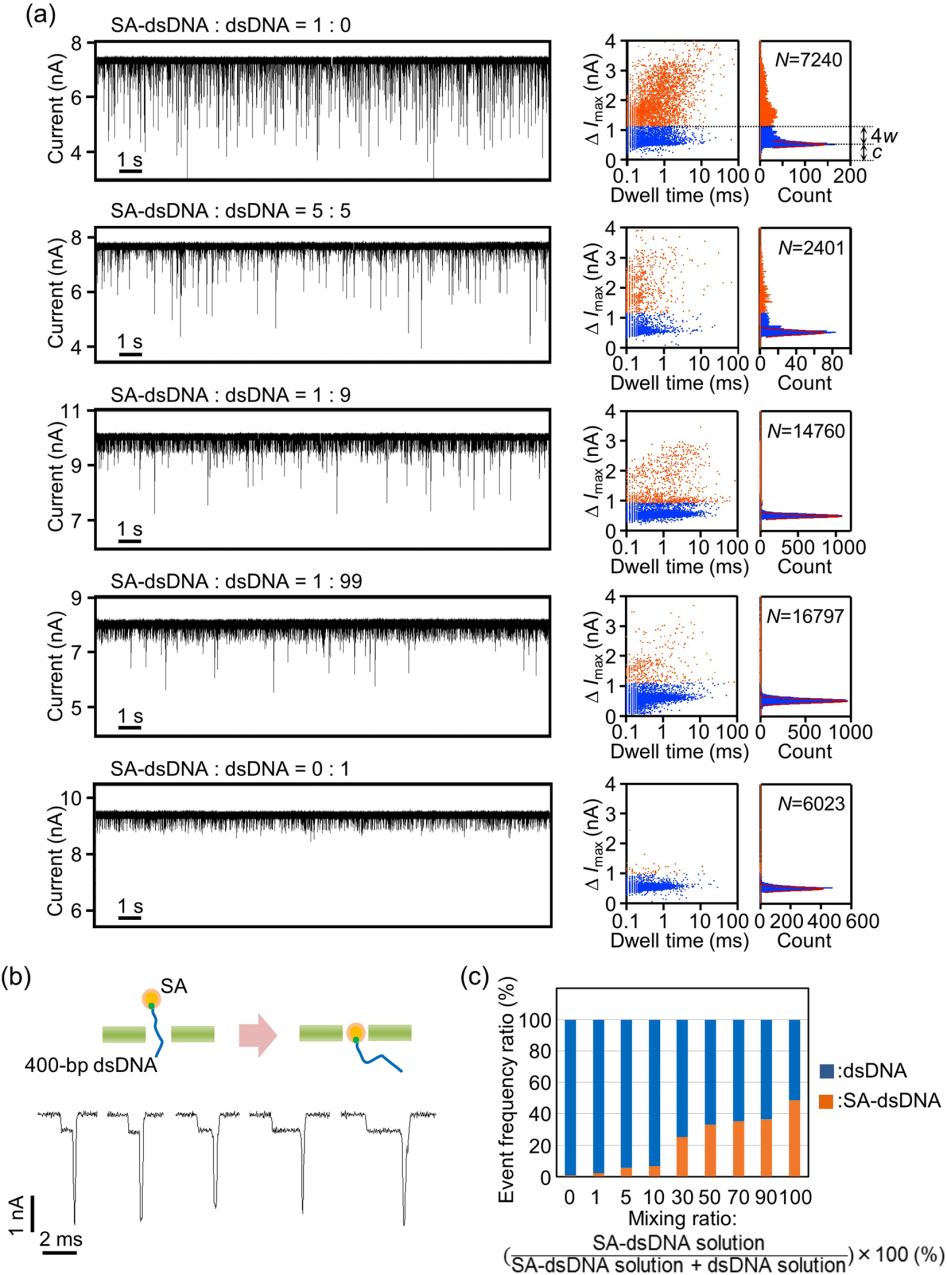
Analysis of current-blockade events derived from SA-labelled and non-labelled dsDNA passing through nanopores at various mixing ratios of SA-labelled and non-labelled dsDNA. (**a**) Time traces of ionic current through nanopores fabricated in 14-nm-thick SiN membranes, scatter plots of current-blockade events and histograms of Δ*I*_max_. The voltage applied was 0.2 V (*V*_trans_ = 0.2 V and *V*_cis_ = 0 V). Among nine experiments, the results for five different mixing ratios of SA-labelled 400-bp dsDNA and 400-bp dsDNA are shown. The estimated diameters of the nanopores were 8.8 nm, 9.1 nm, 10.8 nm, 9.4 nm and 10.4 nm in order from the top to the bottom. (**b**) Magnified views of typical ionic-current blockades caused by SA-labelled 400-bp dsDNA passing through a nanopore. (**c**) Frequency ratios of current-blockade events derived from SA-labelled and non-labelled dsDNA passing through nanopores at nine different mixing ratios of SA-labelled and non-labelled dsDNA.

Next, we examined the event discrimination of SA-labelled and non-labelled 400-bp dsDNA passing through nanopores fabricated in 14-nm-thick SiN membranes (Fig. 8). Nine different aqueous solutions for the *cis* chamber were prepared; that is, a solution of 1 M KCl with 15 nM SA-labelled 400-bp dsDNA and a solution of 1 M KCl with 15 nM 400-bp dsDNA were mixed at ratios of 1:0, 9:1, 7:3. 5:5, 3:7, 1:9, 5:95, 1:99 and 0:1. Figure 8(a) presents five results from the nine experiments. Compared to the results shown in Fig. 7(h,j), these scatter plots and Δ*I*_max_ histograms exhibit less clear boundaries between the two events derived from SA-labelled and non-labelled dsDNA. We cannot currently provide a clear explanation for this difference. Possible causes include difference in the thicknesses of the SiN membranes (20 nm or 14 nm) and lengths of dsDNA (80 bp or 400 bp). We provisionally determined the boundary value of Δ*I*_max_ derived from SA-labelled and non-labelled dsDNA as *c* + 4*w*, where *c* is the central value of a Gaussian curve fitted to each spectrum around Δ*I*_max_ = 0.5 nA, which was attributed to the events mostly caused by dsDNA translocation through the nanopore, and *w* is the value of the full width at half maximum (FWHM) of the Gaussian curve.

Small current blockades derived from dsDNA translocation through the nanopore were observed even in the case of SA-labelled dsDNA: dsDNA = 1: 0 due to the remaining dsDNA unreacted with SA. On the other hand, large current blockades derived from SA-labelled dsDNA translocation through the nanopore could be clearly observed even in the case of SA-labelled dsDNA: dsDNA = 1: 99. Figure 8(b) presents magnified views of the typical current blockades derived from SA-labelled 400-bp dsDNA. Each waveform consists of two current-blockade levels, which were not observed when SA-labelled 80-bp dsDNA passed through the nanopore (Fig. 7(f)). Each of the two levels is believed to be attributable to the partial entry of dsDNA or SA into the nanopore. Figure 8(c) presents the frequency ratio of the current-blockade events derived from SA-labelled and non-labelled dsDNA in each of the nine experiments. The figure confirms that the event frequency ratio changed, reflecting the mixing ratio of the two solutions containing SA-labelled dsDNA and dsDNA.

## Discussion

Stable fabrication of a single large nanopore in a SiN membrane by CBD was examined. First, we applied CBD to 20-nm-thick SiN membranes under various pH conditions. Instead of a nanopore, a local conductive-film portion was created in the membrane in the case of pH < 11.3. On the other hand, a single nanopore was fabricated when pH ≥ 11.3. This result suggests that the membrane could be etched to form a nanopore by hydroxide ions under a high electric field. The conduction-ion distribution in the conductive-film portion, incidentally, could be extrapolated from the shape of the *I*-*V* curve, which changed depending on the pH of the aqueous solution.

The size of the nanopore could be controlled by changing the applied voltage or *I*_cutoff_ during CBD at a pH of 12.7. For example, nanopores with diameters of 5 to 40 nm could be fabricated by changing the value of *I*_cutoff_ from 0.1 μA to 6.5 μA at an applied voltage of 18 V. The effective thickness of the fabricated nanopores was found to be approximately 12.5 nm, which was estimated from the relationship between the conductance (*G*) and equivalent diameter (*d*_TEM_) of the nanopores. In addition, this high-pH CBD method was also effective in nanopore fabrication in 14-nm-thick SiN membranes. Nanopores with diameters of 7 to 12 nm could be fabricated by changing *I*_cutoff_ from 0.3 μA to 1 μA at an applied voltage of 11 V, and the effective thickness of their nanopores was found to be approximately 11 nm.

The occurrences of SA-labelled and non-labelled dsDNA passing through the fabricated nanopores could be distinguished by the difference in ionic-current blockades caused by those two molecules. The ionic-current blockades derived from SA-labelled dsDNA translocation through the nanopore could be clearly detected even when the concentration of SA-labelled dsDNA was only 1% of the total concentration of dsDNA. In addition, the frequency ratio of the current-blockade events caused by SA-labelled and non-labelled dsDNA changed, reflecting the mixing ratio of SA-labelled dsDNA and dsDNA.

The method of CBD under high-pH conditions examined in this study is a simple and rapid (less than 1 min) nanopore fabrication method that enables the stable fabrication of various-sized solid-state nanopores without the need for expensive equipment such as EB lithography, TEM or helium-ion microscopy (HIM). In addition, the fabricated nanopores showed good performance in the detection of different-sized molecules. Consequently, we believe that this nanopore fabrication method will accelerate the research and development of nanopore devices for detecting various-sized molecules.

## Methods

### Fabrication of membranes

The SiN membranes were fabricated on an 8-inch silicon wafer with a thickness of 725 μm. The fabrication process was the same as that described in our previous report^56^. First, a SiN layer with a thickness of 20 nm or 14 nm was deposited on both sides of the Si wafer via low-pressure chemical vapour deposition (reacting gases: SiH_2_Cl_2_-NH_3_; flow ratio: SiH_2_Cl_2_:NH_3_ = 1:25; 770 °C; 0.5 Torr). The composition ratio of the SiN layer analysed by X-ray photoelectron spectroscopy (PHI 5000 VersaProbe II, X-ray: Al Kα, ULVAC-PHI, Inc.) was approximately Si:N = 1:1.24. Then, a SiO_2_ sacrificial layer with a thickness of approximately 260 nm was deposited on the SiN layer on the front side of the wafer, and a SiN layer with a thickness of approximately 90 nm was deposited on both sides of the wafer. Next, the top SiN layer in each square area approximately 600 × 600 nm^2^ in size and the backside SiN layer in each corresponding 1038 × 1038 μm^2^ square area were subsequently etched by reactive-ion etching, followed by etching of the Si substrate with tetramethylammonium hydroxide (TMAH) at 85 °C for approximately 9 hours. After etching of the Si substrate, the wafer was diced into chips. Finally, the SiO_2_ sacrificial layer was etched with potassium hydroxide (33 wt.% solution of KOH at approximately 70 °C) before the dielectric breakdown experiment of the SiN membrane.

### Observation of SiN membranes

Observations of the SiN membranes were performed using a field-emission TEM (JEM-2100F (HRP), 200 kV, JEOL, Ltd.). Before the observations, the membranes were immersed in deionized water for a day to remove any salt residues. The areas of the fabricated nanopores were measured using image processing software (ImageJ, National Institutes of Health, Bethesda, MD, USA). The observation process described above was the same as that described in our previous report^56^.

### Setup for dielectric breakdown experiments

Initially, the SiN membrane was mounted onto a custom-built acrylic flow cell. Two chambers (each with a volume of 90 μL) separated by the membrane were formed in the flow cell: a *cis* chamber and a *trans* chamber. Aqueous solutions at various pH values were prepared by adding KOH or HCl to 1 M potassium chloride, 10 mM Tris-HCl, and 1 mM EDTA. The pH of the aqueous solution was measured using a LAQUA act pH/OPR/COND METER D-74 (HORIBA Scientific, Japan). After filling the aqueous solution into both chambers, two Ag/AgCl electrodes were immersed in both solutions to ensure electrical contact. Application of a constant voltage, measurement of the current through the membrane during CBD, and measurement of the *I-V* characteristics were performed using a 4156B precision semiconductor parameter analyser (Agilent Technologies, Inc.). The cut-off current for CBD was set as the current compliance value of the 4156B system.

### Preparation of streptavidin-labelled 80-bp dsDNA for measurements

First, an 80-mer single-stranded DNA (ssDNA) whose 5′ end was modified with biotin was annealed with a primer. Both the biotin-modified ssDNA and primer were purchased from Sigma Aldrich, Japan. Then, the primer was extended by an elongation reaction, and biotin-modified 80-bp dsDNA was created. The sequences of the ssDNA and the primerwere 5′-ACGAATTCGAGCTCGGTACCCGGGGATCCTCTAGAGTCGACCTGCAGGCATGCAAGCTTGGCACTGGCCGTCGTTTTACA-3′ and 5′-TGTAAAACGACGGCCAGT-3′, respectively. The elongation reaction was performed in 50-μL reaction volumes consisting of 3 μM primer, 3 μM ssDNA, 320 U Bst 3.0 DNA polymerase, 1X isothermal amplification buffer, 1 mM dNTPs and 1 mM MgSO_4_ (New England Biolabs, Ipswich, MA). The time and temperature of the reaction were 60 min and 65 °C, respectively. The product after the elongation reaction was purified using NucleoSpin® Gel and PCR Clean-up (Takara Bio Inc, Japan). The purified product was then quantitated with a Qubit fluorometric system (Life Technologies). A biotin-streptavidin binding reaction was performed in 18.3-μL reaction volumes consisting of 1.5 μM biotin-modified dsDNA and 10 μM streptavidin (Thermo Fisher Scientific, Waltham, MA). The time and temperature of the binding reaction were 37 °C and 30 min, respectively.

### Preparation of streptavidin-labelled 400-bp dsDNA for measurements

A portion of λDNA (NIPPON GENE CO., LTD, Japan) was duplicated by PCR amplification using a biotin-modified forward primer and a biotin-unmodified reverse primer to obtain biotin-modified 400-bp dsDNA. PCR amplification was performed using MiniAmp Plus Thermal Cyclers (Thermo Fisher Scientific, Waltham, MA). Both ssDNA and primer were purchased from Sigma Aldrich, Japan. The sequences of the forward and reverse primers were 5′-TGCAACGAACAGGTCACTATCA-3′ and 5′-GAGCAAAGCAAAACAGGCGTA-3′, respectively. PCR amplification was performed in 50-μL reaction volumes consisting of 0.3 μM forward primer, 0.3 μM reverse primer, 50 ng λDNA, 1 U KOD-Plus- Neo, 1X PCR buffer for KOD -Plus- Neo, 0.2 mM dNTPs, and 1.5 mM MgSO_4_ (TOYOBO CO., LTD., Japan). The cycling parameters of PCR were 94 °C for 2 min → [98 °C for 10 sec → 58 °C for 30 sec → 68 °C for 30 sec] × 35 times. The product obtained after PCR was purified using NucleoSpin® Gel and PCR Clean-up (Takara Bio Inc., Japan). The purified product was then quantitated with a Qubit fluorometric system (Life Technologies). A biotin-streptavidin binding reaction was performed in 18.3-μL reaction volumes consisting of 1.5 μM biotin-modified dsDNA and 10 μM streptavidin. The time and temperature of the binding reaction were 37 °C and 30 min, respectively.

### Setup for measuring DNA translocation through a nanopore

Prior to the measurements of DNA translocation through a nanopore, the aqueous solutions in both chambers were displaced by a 1 M KCl aqueous solution at a pH of 8.7. After that, the solution in the *cis* chamber was displaced by a 1 M KCl aqueous solution containing samples (i.e., SA-labelled dsDNA and dsDNA) at a pH of 8.7. The ionic-current measurements were performed using a patch-clamp amplifier (Axopatch 200B, Axon Instruments, Union City, CA). The detected current was low-pass-filtered with a cut-off frequency of 10 kHz using a four-pole Bessel filter and then digitized with an NI USB-6281 18-bit DAQ AD converter (National Instruments, Austin, TX) at 50 kHz. Finally, the current was recorded on the hard disk of a personal computer. All the measurements described above were performed at room temperature.

## Supplementary information

Supplementary Information
