# Supplementary material for: Stable fabrication of a large nanopore by controlled dielectric breakdown in a high-pH solution for the detection of various-sized molecules
Source: Sci Rep. 2019 Sep 11;9:13143. doi: 10.1038/s41598-019-49622-y (PMC6739384; doi:10.1038/s41598-019-49622-y)
Supplement: Supplementary file 1 — Supplementary Information [file 41598_2019_49622_MOESM1_ESM.doc]

**Supplementary Information for**

**Stable fabrication of a large nanopore by controlled dielectric breakdown in a high-pH solution for the detection of various-sized molecules**

Itaru Yanagi*, Rena Akahori and Ken-ichi Takeda

Hitachi Ltd., Research & Development Group, Center for Technology Innovation - Healthcare, 1-280, Higashi-koigakubo, Kokubunji, Tokyo, 185-8603, Japan

**Supplementary Figure 1. Generation of multiple nanopores by CBD.** (a) Current-time trace during CBD of a 10-nm-thick SiN membrane in a 1 M KCl aqueous solution at a pH of 7.5. The applied voltage was set at 6.5 V, and the cut-off current was set at 0.4 μA. (b) Image of the entire membrane after CBD. Created nanopores are indicated by yellow arrows. The scale bar is 200 nm. (c) Magnified views of the created nanopores. The scale bars are 10 nm.

**Supplementary Figure 2. *I*-*V* characteristics of 20-nm-thick SiN membranes after CBD with *V*_trans_ = 20 V and *V*_cis_ = 0 V.** *I*_cutoff_ was set at 1 μA. *V*_cis_ was set at 0 V during the measurements of the *I*-*V* curves.

**Supplementary Figure 3. *I*-*V* characteristics after CBD under several pH, voltage, and *I*_cutoff_ conditions.** *I*-*V* curves were measured after CBD of 20-nm-thick SiN membranes. *V*_cis_ was set at 0 V during the measurements of the *I*-*V* curves.

**Supplementary Figure 4. Time required to form a nanopore by CBD.** The relationships between *d*_TEM_ and the time required for the current to reach *I*_cutoff_ from the start of CBD. Nanopores were fabricated in (a) 20-nm-thick SiN membranes and (b) 14-nm-thick SiN membranes in a 1 M KCl aqueous solution at a pH of 12.7. The applied voltages were (a) 18 V and (b) 11 V.

**Supplementary Figure 5. Electropherograms of SA-labelled dsDNA and non-labelled dsDNA.** The prepared samples were analysed by gel electrophoresis using an Agilent 4200 TapeStation System (Agilent Technologies, Inc.). D1000 Screen Tape, D1000 Reagents and D1000 Ladder were used in the analyses. (a) Electropherogram of SA-labelled 80-bp dsDNA and non-labelled 80-bp dsDNA. (b) Electropherogram of SA-labelled 400-bp dsDNA and non-labelled 400-bp dsDNA. Band shifts caused by the binding of SA and dsDNA were clearly observed.
